# Sketch of the Medical Topography of Malta

**Published:** 1824-12

**Authors:** 

**Affiliations:** Deputy Inspector of Hospitals; Author of the "History of the Plague of Malta."


ART. IV.—*Sketch of the Medical Topography of Malta.* By —— TULLY, Esq. Deputy Inspector of Hospitals; Author of the "History of the Plague of Malta."

THE Island of Malta rises nearly in the centre of the Mediterranean, between the fifteenth and sixteenth degrees of east longitude, and between the thirty-fifth and thirty-sixth degrees of north latitude. The island is every where extremely narrow, its greatest width not exceeding ten miles, and we find its circumference only marked at sixty miles. What the original extent of the island was, or whether it formed a part of the continent of Africa, is a question which has frequently attracted the notice of the historian, and one which, in all probability, will for ever remain involved in obscurity: certain, however, it is, that at some period the island must have been of greater extent than at the present day, as vestiges of roads are now distinctly traceable to the verge of the precipices overhanging the sea, to the southward of the island, and that point lying opposite to the coast of Africa; and the neighbourhood of these precipices is the only place where a landing is not practicable, the coast every where else being easy of access. There are circumstances, when considered, that lead us to conjecture, that not only Malta, but even Sicily, formed part of the continent, and having been separated from it by some extraordinary convulsion of nature: for, on examining the strata of Malta, we find its direction to lie, as near as possible, SSW. by compass, forming a direct line, and leaning towards the opposite land of Africa; whilst the opposite point would, in a continued line, touch Cape Passaro in Sicily. This direction corresponds, in some respects, with Grenough's statement, who, in his "Geology," tells us, that the strata of Syracuse agrees with that of Malta; and brings forward other reasons, foreign to our purpose, for believing that Malta had formerly been joined to a high land.

The land of Malta is throughout extremely low; the greatest point of elevation (which we find to be St. George's, in the neighbourhood of the Bochetta,) not rising 1200 feet above the level of the sea; and the next highest range sufficing only to facilitate the conveyance of the water of the aqueduct to the capital; and these ranges, as far as I can judge, have a claim to the primitive order. Indeed, so low does the land appear, that, upon approaching the island, under the most favourable circumstances of weather, it is not discernible until you arrive within five and twenty miles of the shore.

The appearance of the land is by no means prepossessing,—scarcely gratifying even to the sea-sick traveller; as it offers to the eye little more than a succession of broken calcareous rock, leading, upon the first view, to the idea of the whole surface having been artificially prepared, and ready for the lime-kiln.

This appearance of the general face of the country is chiefly owing to the peculiar nature and undulating form of the ground,—a large portion of the whole of the territory of the island being partitioned into small terraced plots; a precaution in cultivation, in Malta, indispensibly necessary; without which, much of the soil would be in danger, both from the looseness of its texture and its great lightness, of being washed away by the heavy rains that frequently fall during the spring and autumnal months. From this barren and uninteresting view, our attention is at once attracted to the superb harbour of Malta, almost every where crowned with majestic buildings: the city of Valetta, with its suburb Floricanea and Fort St. Elmo, on the right; and, on the left and continuous line, the outlying fortifications of Ricasoli, St. Angelo, and the three cities, Vittoriosa, Burruch, and Singlia. Besides these principal residences, Citta Vecchia deserves to be named, although in the interior of the country,—not only from its extent and situation, which stands upon the highest habitable part of the island, but from its having been previously the capital of the island. Independent of these four cities, we observe twenty-two *casels*, or small towns, dispersed throughout the island, affording ample accommodation for the inhabitants.

The situation of Valetta, the capital of Malta, is peculiarly happy, both for health and commerce, placed as it is upon a peninsula, having on either side an excellent harbour; whilst its southern extremity is washed by the sea. The houses in Valetta (and, generally speaking, those throughout the island,) are large and commodious: the rooms are of a height unknown in our own country, and the windows are proportionately large; the walls are extremely massy, being from three to five, and even six, feet wide; rendering them, upon the whole, admirably adapted to the climate. These buildings are formed of a soft free-stone, portions of the rock of which the island is composed; and, from the great facilities which its peculiar softness offers to the workman, it is readily moulded into all shapes; hence the grandeur and magnificence of the public edifices, and most of the residences of the nobility of the island.

The residences of the poorer classes, where our attention in medical topography becomes more particularly directed, are, not only in Valetta, but throughout the island, as little liable to exception, both in point of dryness and ventilation, as any country with which I am acquainted: indeed, generally speaking, they are airy and comfortable; and, though many of them in the cities lie below the level of the neighbouring streets, and others, as in every other country, in cellars, nevertheless their interiors do not, as far as I have seen, offer those causes for insalubrity or disease, so generally met with amidst crowded populations, and discovered amongst the lower classes of society.

Valetta, and the principal towns above enumerated, are every where shut in by immense fortifications, which the successors of the great founder of the capital, whose name it bears, endeavouring to emulate his example, have greatly multiplied; but we find (contrary almost to a general rule) that these stupendous works rather contribute to, than detract from, the health of the inhabitants within their respective lines, as the prodigious depth, as well as the great width, of these ditches favour a free circulation of air; whilst the extent of the various outworks and glacis prevent the approach of crowded residences. Besides, these ditches being for the greater part cut through the solid rock, and no place, in any direction, left for the lodgment of water, or soil for the production of vegetable matter, (with the exception of a few spots, converted, with much attention and great labour, into vegetable gardens,) the evils generally attendant upon fortified towns, particularly in warm countries, are, from these causes, entirely avoided.

The principal, and, in fact, (as far as I can discover) the only evil which Valetta laboured under after its first figuring as a capital, was its precarious supply of water; but an element, upon which the health of the population so much depended, soon, under a provident government, became an object of a peculiarly important consideration. We find, upon the building of the city, that each house was provided with a private and spacious cistern: these cisterns were arranged with great care, and proportioned to the size of the dwelling, the ordinary number of inmates usually calculated upon for each residence, and mostly for a supply for two years. These cisterns were, and continue to be, supplied with rain water, conveyed chiefly, by numerous conductors, from the roof of each dwelling, which we find universally flat in the island, no doubt for the purpose of collecting water. But these precautions were found insufficient; and, for the better supplying the town, several public cisterns and fountains were built; the former chiefly within the forts and military quarters, and the latter dispersed through the town. These were supplied by means of an aqueduct, built after an infinity of labour and expence, conveying excellent spring-water upwards of 17,000 English yards; the advantage of which is still felt. Upon the completion of this aqueduct, many private houses were permitted to establish pipes of communication; by means of which, in times of scarcity of water, they may be supplied from the public reservoirs.

In no modern town, with which I am acquainted, has more attention been paid to the construction of public sewers, than in the capital of Malta. The town was built in the flourishing days of the Order, when it is evident that no expence was spared to execute a plan, which, as far as local situation would permit, was perfect in all its branches. The plan of the sewers appears to me to have been taken from those of Rome, constructed in the days of her greatness. I need not add, that, in regard to the relative size, an enormous difference exists; but I mean to be understood, that the principle upon which they are formed is one and the same. In Valetta, we find that every street is furnished with a deep and spacious sewer, or rather a canal, upon an inclined plane, sufficient to prevent any great lodgments of filth; and these are every where so secured and covered in, as to prevent most effectually the escape of offensive effluvia. All these canals have a free communication with the sea, and are so constructed that the least defect can be immediately detected, and remedied without public nuisance. Each house has a direct communication with a sewer, and dirty water, water from washing (which is chiefly done in town by the natives), rushes, by means of house-drains, into the sewers, carrying with it the daily foul accumulations, to the regular reservoir; from whence, by its specific gravity and the nature of the ground it passes, it is constantly propelling itself forward to its natural outlet; when, intermixing with the sea, no evil can ensue. Hence, although there is no fall of rain frequently for six months in succession, no inconvenience whatever is felt; nor can the nicest sense of smell detect the existence of these conductors in the streets; and this in a climate and at seasons peculiarly adapted to the development of noxious effluvia.

We are not here to confound with this statement the effluvia which, in every town and in every country, must arise, and be perceptible, within the immediate sphere of public necessaries; but even this I consider to be as little felt in Valetta as in towns situated in much milder degrees of latitude. These sewers are, as may be concluded, thoroughly cleansed annually by the successive columns of water that sweep through them during the rainy seasons.

Previous to the plague, all ranks were buried within the churches; but, since that period, this has been confined to the higher and middling classes, whilst the lower orders, both of towns and casels, are interred in appropriate places, removed from the public residences, carefully walled in. There is a separate burial-place for the British: the situation is well adapted within the Florian lines, but, as the ground is newly made, and by no means close or compact, (having been formed from the debris of the neighbouring land, it requires occasional inspection, that the grave should be deep; and, when vaults are not used, according to the different seasons, quick lime ought to be mixed with the covering ground. I notice these particulars, having been called upon, some time since, to report upon the subject to government. Notwithstanding the frequency of the custom of burying within the churches, it does not appear that any evil has arisen from a practice, which is held up as so dangerous in hot countries.

The principal buildings in Valetta are the palace,* the hotels formerly the residence of the various knights of the Order, and now for the most part occupied as military quarters, the Treasury, the Palace of Justice, the church of St. John, the library, the hospitals, both civil and military, and the barracks.

To enter upon a minute description of the public buildings, I conceive to be entirely foreign to the views of the medical reporter, particularly as they can only refer to those periods when the fine arts were more happily cultivated in the country. The exterior of most of these buildings are in a high state of preservation; some of them, as the Auberge de Castille, being, as far as my slender knowledge of the matter leads me, perfect models for the architect.

It is a current, but very erroneous, opinion, that Malta is little more than a barren rock; and certainly, as I have stated, the first appearance of the island would lead to the belief: and it was also an opinion, which received some credit from the writings of Brydone, that such was at one period the nakedness of the rock, that it had been dependent upon Sicily for the soil which afforded its inhabitants the means of subsistence. But a more intimate knowledge of the country than what Brydone appears to have had, satisfies us of the fallacy of the assertion; and I am inclined to believe that our author, in giving his opinion upon the subject, followed the general belief of the time he wrote. In the first instance, in no part of the island do we meet with a soil similar to the heavy and rich loam of Sicily; and in every part, save the southern extremity in the African line, (where the soil is the red argillaceous,) the soil is the

* Some idea may be formed of the size of this structure, when we state that it was upwards of five years building, although a prodigious number of persons were employed,—a number frequently exceeding six hundred.

same, and evidently native; for we find it to consist of clay, largely intermixed with a sand, or gravel, corresponding with the bed of rock over which it is laid, and the greater part of the soil evidently formed by the same process,—a process which may almost be daily noticed in one part or another of the country, as patches of the rock are constantly under the hammer, and worked for the purpose of cultivation. The very superficial tenure of the greatest part of the soil of the island is almost proverbial amongst the inhabitants, and is one of the principal causes to which we may attribute the great salubrity of the air; and to which we might add the porous texture of the rocky foundation, ready to absorb all superfluous moisture. The mode of what the peasant terms making the soil, is necessary to be understood, as its quality cannot detract from, but materially tends to a pure atmosphere. The following is the plan I have seen adopted, and that which I believe is the general practice:

The rocky ground intended to be reclaimed is first cleared of all loose stones and earthy substances, which may be scantily lying on the cliffs and interstices of the rock: these are carefully heaped together on the most level spot of the rocky surface about to be worked. The labour then commences, which consists in breaking down the rock into fragments; a task of no small difficulty,—for, although the stone is particularly soft, yet the external surface, exposed to the action of the air, is hard, requiring in the onset a very large share of labour. The breaking-up of the rock is persevered in until the whole becomes level. In this manner they proceed until a flat is formed of about half an acre, which is occasionally propped in various parts by massy stone walls; a work which entirely depends upon the inequality of the ground, and thus arranged for the reasons already mentioned. In the general process, there is seldom less than three feet horizontally broken of the rock, when a thin layer of earth is, for the most part, discovered. If the layer at this point is not complete, the rock is generally found full of crevices of various sizes, as if it had been acted upon by some external agent, and these crevices are invariably found filled with a rich clay. Here the great labour terminates, and the whole is now, with great industry and care, mixed together.

What is thus denominated "new-made ground," and which, as we have shown, mostly consists of broken-down free-stone, very early offers proofs of fertility; and, from its great aptitude, in its new form, to the absorption of moisture from the atmosphere, its bulk very perceptibly increases, and soon forms a sort of concrete texture. Those plants which require least nourishment, as the cucumber and water-melon, are the first reared, and are generally found to flourish the succeeding season. The products next in succession that are reared, demand, like the former, no great nourishment from the newly-made soil. Thus, with the occasional aid of small portions of manure, and the vegetable remains, which are allowed to decay upon the ground, a daily improvement is observed,—so much so, that corn is usually the growth of the third year; and it is by this and similar processes, that a large portion of the ground of Malta has been brought to agricultural purposes, and by subsequent care, aided by climate, rendered extremely fertile.* Indeed, such is the fertility of the soil, that the common observation is, that it is always producing.

From what has been said of the soil, the implements of agriculture will readily be believed to be, what they truly are, extremely simple; nor will it be expected that much labour is necessary in ploughing: in fact, a light plough, worked by an ox and a mule, is generally sufficient,—always, when the ground to be ploughed is level. The hoe is the other principal implement of the husbandman.

We find the general produce to be wheat, barley, oats, Indian corn, cotton, cinnamon, aniseed, lichen,† hay, and a species of clover called silla,‡ which is indigenous; to which we may add, almost every species of fruit and vegetables, and both one and the other, particularly the latter, in great abundance and tolerably good. The nature of the soil, as well as the great value of land, renders it inapplicable to pasture: there is, therefore, very little of it used for this purpose; consequently, most of the cattle, particularly the larger class, are stall-fed.

Among the various products, the cotton is the most esteemed, forming not only the staple commodity, but likewise an article of great profit, no part of the plant being without its use: the seed, formed into cakes, is the chief food of the larger cattle, which makes the beef of Malta so much esteemed; whilst the leaves afford nourishment to the smaller animals, as sheep and goats: with the latter of which the island abounds, being of an excellent species, yielding immense quantities of milk, and of a good quality.

We have now seen how much the soil of this island must contribute to the general health of the inhabitants, and, upon fur-

---

* Notwithstanding the fertility of the soil of Malta, the inhabitants are indebted to foreign countries for a large portion of the common necessaries of life. The island only grows corn for six months; the rest is imported from the Black Sea and Egypt; wine and oil from Sicily, and other parts of the Mediterranean.

† The lichen is used as a dye.

‡ The silla is denominated, by Tournefort, *hedysarum clypeatum flore suaviter rubente.* It grows from three to four feet high; the colour is beautiful; it is extremely luxuriant in its growth, and gives an exceeding rich appearance to the ground where it grows; and is in full bloom the latter end of March.

ther examination, we shall find the influence it produces upon the atmosphere: for, admitting the peculiarly absorbing quality of the soil, it necessarily follows that the atmosphere must at all seasons contain a very diminished quantity of vapour, compared to countries enriched by a heavier and more retaining earth, giving advantages, from this effect alone, to the climate of Malta, and rendering it thereby more adapted to health than the climates of the surrounding states. That this is the case, experience also proves: nevertheless, as the subject is one of importance, it behoves us clearly to point out the actual superiority which the island possesses, and how free it is from the general causes of insalubrity that mark the neighbouring countries.

In the neighbouring islands and continents, where marsh is prodigiously fertile, the health of the inhabitants in their vicinity is found constantly to change with the seasons; and where marsh predominates, and is aided by these particular constitutions of the air, which occasionally spread their influence over immense tracts of land, those epidemic diseases arise, that are become so common a scourge to the human race; and the severity of the reigning disease is felt according to the nature and extent of the predisposing causes. Now, as regards Malta, the first and best proof we can offer in favour of the climate, is the absence of all epidemic diseases; and the next, as we have shown, the want of material for the formation of marsh. However, in stating thus much, I am quite aware that a different opinion is maintained by some; and that the plague of 1812 is even considered as an epidemic visitation. Dr. Hancocke, in a late work upon the "Laws of Contagion," certainly wishes to impress this opinion upon the public; and he is equally desirous that it should be believed (as it falls in with his own theory) that the island is no stranger to marsh, without which, much of his ingenious argument would necessarily fall to the ground; but that this is not the case, I think I shall be fully able to prove: at all events, if any thing of such a nature is found to exist, it must take a more appropriate term than that of marsh,—unless, indeed, that my ideas of marsh are proved to be erroneous.

In speaking of marshy countries, our ideas naturally become associated with the term in its received interpretation, or what we ourselves are accustomed to consider as marsh: hence, we conclude that large tracts of uncultivated land, rich in soil, and in vegetable matter in a constant state of decay, abundantly supplied with moisture in undrained portions, constitute what is generally understood as marsh; and, according to the different degrees of solar heat, shall these exhalations arise, that, combined with other causes, as yet very imperfectly known, will produce those evils above noticed, and to which countries variously situated are exposed, producing, according to climate, those varieties of fever included by nosologists under the different heads of intermittent and remittent, in all their various forms, from the common quotidian up to the yellow fever. This is my opinion of what marsh and its consequences are; and, in adverting to the name, the mind naturally dwells upon what we are wont to denominate as such; as the Pontine marshes in the Roman States, the Lentine swamps in Sicily, the extensive marshy flats of Bucristro, those of the Venetian States, and the extensive fens of England, those never-failing sources of ague; and, to bring the matter closer to this place, the marshes of Catalonia, Santa Maura, and Corfu, with numerous others on the Mediterranean shores. But surely we are not authorised to designate a country as marshy, from which so many inferences may be drawn, and upon which so many false theories may be built, because deleterious exhalations (as we usually express it) may mark one or two spots of a territory, and because a few cases of intermittent and remittent fevers may annually be noticed amongst a population amounting to upwards of a hundred thousand souls. The truth is, there is no country, however happily situated, where some local causes will not be found to co-operate to produce diseases of this character, particularly in the countries exposed to high atmospheric temperature: thus, in some places, uncleansed ditches and blocked-up drains, crammed with decaying vegetables; and, in others, large open wells, used for watering vegetable-grounds, (as we frequently meet with in Italy and Sicily,) in which the vegetables are dipped for the purpose of freeing them from their outward leaves, previous to their being sent to market, and where, from want of attention, they are allowed to float upon the surface frequently in large quantities, and from which, like the former, exhalations arise during the summer-months, capable of producing both remittent and intermittent fevers amongst those residing in the immediate vicinity, as I have frequently had occasion to observe: and I may assume, that I do not go beyond the bounds of reason, when I say, that our very kitchens, if they are permitted to retain vegetable matter in a state of decomposition, may, under certain limitations, produce the effects of the worst marshes upon those exposed to such peculiar miasma; and it will be found that these various causes, with many others of a similar nature, will, as I have above stated, frequently exist in the healthiest countries. It is not, therefore, because fevers of a particular type are occasionally to be met with in the island, that we are to conclude that they are necessarily the offspring of marsh. We know to the contrary; and, if we except the Marsa, presently to be noticed, the causes of these fevers, wherever they do exist in the island, must be traced to one or other of the sources just mentioned; whilst one of the chief predisposing causes will be discovered to depend upon the want of proper aliment, as we find these diseases only traceable to the poorer classes of society; and that this is the case, our knowledge of the country, and its inhabitants, warrants us to assert.

Dr. Faulkner is the last author who has given us a "Sketch of the Medical Topography of Malta;" and so cautious is this writer upon the subject of marsh, as connected with the island, that he says "there are only *three spots* upon the habitable part of the rock which can deserve to be called marshy, or even moist, —the Marsa, Messida, and Puales." In my "History of the Plague of Malta," I have noticed the Marsa alone, from the conviction that neither the Messida nor Puales were worthy of being named; nor would I have entered upon any explanation of the nature of either one or the other, had not this difference in our statements been noticed by Dr. Hancocke, who, from a feeling too evident, gives the credit of correctness of statement to Dr. Faulkner; although, were I even to admit that these marshes did really exist, they could have but little weight, indeed, in bearing the learned author out in his assertions. But, as the subject is now some time before the public, I think it as well not to pass it entirely unnoticed.

It appears to me that the ground called La Marsa derived its evil from far different causes to what its name has been erroneously supposed to imply,* being nothing more than a piece of level land at the head of the main harbour, from whence the sea, in the course of years, has been gradually receding, and as gradually filling up by alluvial deposits from the neighbouring high grounds; and which, to this day, is subject to inundations of the sea in that part yet unfilled, during the prevalence of the north-east winds, that blow directly into the harbour, rolling its sea onwards, uninterrupted, from the Adriatic. That such is the nature of the ground, we have the most undeniable proof; as the sea was known, within the memory of many inhabitants, to have covered the fine cultivated vale that runs from the Marsa close to Casal Curine.

The heads of all harbours that run into shallows, and particularly those shallows that are occasioned by the natural tendency, on particular shores, to the receding of the sea, become (as far as my experience goes), under certain circumstances, to

* The term *Marsa* has been generally considered to imply Marsh, when, in point of fact, it means Port, being the Arabic for port, or rather anchorage; and is likewise applied to all the principal ports in the island,—as Marsa Mucetto, Marsa Skalli, and Marsa Sirocco. This is particularly noticed by Fiancesco Correggio, in a work on Malta, in the Spanish language, printed at Barcelona in the year 1568.

the full as fertile sources of disease, in warm climates, as the best-formed swamps, producing all those alterations in the surrounding atmosphere necessary for the production of the most fatal fevers; owing, as we may presume, to the rapid decomposition, in the appropriate seasons, of the marine matter constantly thrown upon these flats, from the occasional rising, as well as from the ordinary agitation, of the sea, leaving, upon the subsiding of the water, larger deposits in some places than in others: and this we find to be the case in the ground we allude to; nor can it be expected that these causes will entirely cease in their effect, until they are totally removed either by nature or by art. The former by the gradual, but certain, receding of the waters in all those low situations where it is once observed to commence, and by the subsequent filling-up of the whole by alluvial deposits; a process more readily effected in countries subject to sudden and heavy falls of rain after long droughts; and the latter by well-formed embankments, by which means the ground, thus defended against the future inroads of the sea, will, after a certain lapse of time, whether cultivated or otherwise, be entirely freed from all offensive matter, and the surrounding atmosphere, by the removal of the cause, be restored to health.

It is to the former of these causes, as I have elsewhere said, that we must attribute the evils of the Marsa, as well as the subsequent amelioration of the air, and the decline of what was denominated the marsh; and, if the same operations of nature proceed undisturbed, we may look forward, without any great lapse of time, to the whole being under cultivation; but, as the territory of Malta bears but a small proportion to its population, in all probability these tardy operations will be superseded by the industry attendant upon an over-stocked country. Indeed, we already observe that daily exertions are made to reclaim the remaining space. But, even admitting that these sea-swamps, as we may term them, were more general, I conceive that, from their peculiar character, they must be viewed in a very different light to those natural swamps, the unhappy inheritance of many countries: for, in the first instance, they, for the most part, cover but a very limited extent, and are seldom permanent; and, in the next place, they are generally capable of being removed when they are found injurious, from their vicinity to habitable places. Besides, in all cases which have come within my own knowledge, although the effluvia from these grounds was proved to be most destructive, yet the sphere of its action, unlike the miasmata of marshes, was extremely limited, scarcely exceeding its own boundaries.

We shall now proceed to examine the marsh of Missida, which will not occupy much of our attention. It appears, then, that many years back the head of the Marsa Muscetto, or quarantine harbour, offered a similar aspect to that of the Marsa, although not to the same extent; and that, by the united operations of those causes we have just stated, the whole has been filled up, and either cultivated or built upon, with the exception of a small space kept as a fish-pond, for the supply of small fish in times of scarcity: and this is, bona fide, what has been denominated the marsh of Missida, and one of the hydras of the non-contagionists of plague; and another melancholy proof how far deep-rooted prejudice may lead us. Of this marsh I shall only say, that, as if it were purposely to detract even from its right to the name of a stagnant pool, we find a stream of excellent spring water entering it, and flowing onwards into the sea, to which it is quite contiguous; and this pond is also frequently refreshed by sea water, purposely arranged.*

Having noticed these two points of importance in the medical topography of the island, we now come to the last ground to be described—Puales; no more entitled, in my opinion, to the name of marsh than the preceding. The valley of Puales, situated at the north-western extremity of the island, is one of the most fertile and best cultivated pieces of ground about Malta: it is bounded, at either side of its inland extremity, by rising grounds, in an equal state of cultivation; these grounds gently sloping until they are gradually lost in forming the vale, which from thence continues in a level form, until it terminates at the water's edge; where a solid wall, about six feet in height, is run across, to prevent the encroachment of the sea. The situation of this vale, at the extremity of St. Paul's Bay, from which it takes its name, is extremely interesting, and particularly advantageous to the husbandman; as we observe that the neighbouring rising grounds are enriched by several springs of excellent water. These springs, after watering the adjacent fields, (for which purpose the water is turned off in various directions,) continuing their course along the low grounds, is collected by means of artificial cuts, that conduct the water to the sea, through openings purposely made in the wall. It is the ground through which these cuts (or, more correctly speaking, drains) are made, that I presume has been noticed as marsh; as it is the only spot in the neighbourhood where I could discover any lodgment for water: and, from an accurate examination of the ground in question, aided by Surgeon Fiddes, of the 85th regiment, I feel little hesitation in saying, that the very worst part of Puales bears no affinity whatever to a marshy

* So long back as the year 1772, this spot was considered nearly in the same point of view. Misida: Peschiera, o picciolo ricetto d'acque salmastre sorgenti, che servano a curare, ed ammollire j Lini, e le tele: dove, da poco tempo in quâ, si conservano Pesci, che vi si racchindono."—Ciantar, lib. i. not. i. p. 121.

soil. The vale of Puales has been noticed as being particularly well cultivated, and we trace this cultivation, on the north-western side, to the very wall of embankment; whilst the remaining and only space which has not been apparently for some time under the plough, occupies, to the best of my judgment, somewhat about three English acres: and it is through this uncultivated spot the four drains have been cut for the purposes already mentioned; and we find that not one of these drains is formidable from size, as it did not appear to me that the largest exceeded eighty yards in length, and the width of the broadest was certainly not more than a stepping distance. As to the ground itself, it is what I would call a good firm sod, (for, in cantering over it, little more than the impression of the horses' hoofs were observed,) without a single shoot or rush to impede the foot; having much more the appearance of a moist grass-plat, closely eaten down, than that of a marsh. It should not pass unnoticed, that this ground was visited by us in the month of December, when, of course, it must have been much more moist than during the parching months of summer and autumn. It is to be observed, nevertheless, that (according to the best information) the few inhabitants in the neighbourhood of Puales have, for years past, been subject annually to attacks both of remittent and intermittent fevers; and it may reasonably be supposed that it is owing to these occasional occurrences, that the formidable term of marsh had been applied to this ground, and most likely because it was the readiest mode of accounting for these febrile attacks, as well as the least liable to objection. To the sufferer it matters little what the disease is occasioned by, so long as it continues to be produced; and it is probable that a minute investigation of the real causes of disease amidst the scattered peasantry, brought together under particular circumstances, would never have taken place, but continue to be imputed to the same cause, had it not been for the doubts raised upon the subject by Dr. Hancock.

[To be continued.]

---

